# Posttraumatic Stress Disorder after Vaginal Delivery at Primiparous Women

**DOI:** 10.1038/srep27554

**Published:** 2016-06-08

**Authors:** Maja Milosavljevic, Dusica Lecic Tosevski, Ivan Soldatovic, Olivera Vukovic, Cedo Miljevic, Amir Peljto, Milutin Kostic, Miranda Olff

**Affiliations:** 1 Institute of Mental Health, Palmoticeva 37, Belgrade, Serbia; 2 School of Medicine, University of Belgrade, Serbia; 3 Serbian Academy of Sciences and Arts, Belgrade, Serbia; 4 Department of Psychiatry, Academic Medical Centre at the University of Amsterdam & Arq Psychotrauma Expert Group–Diemen, Netherlands

## Abstract

Although severe gynaecological pathology during delivery and negative outcome have been shown to be related with posttraumatic stress disorder (PTSD) little is known about traumatic experiences following regular delivery, at the expected time and with a healthy child. The objective of our study was to determine the prevalence of PTSD during postpartum period after vaginal delivery and its risk factors. The sample included 126 primiparous women. Monthly, for the next three months, the women were assessed for PTSD using the gold standard interview for PTSD, Clinician-Administered PTSD Scale (CAPS). Risk factors were assessed including sociodemographic variables, personal medical history and clinical variables. After the first month, 2.4% women had acute full PTSD and another 9.5% had clinically significant level of PTSD symptoms. Following the second and the third month, partial PTSD was found in 5.9% and 1.3% of the women, respectively, and none of participants had full PTSD. Obstetrical interventions were the only significant risk factor for the development of PTSD. Symptoms of postpartum PTSD are not rare after a traumatic delivery, and associated with specific obstetrical risk factors. Awareness of these risk factors may stimulate interventions to prevent this important and neglected postpartum disorder.

Posttraumatic stress disorder (PTSD) is characterized by symptoms of re-experiencing, avoidance and hyperarousal, as well as social and professional dysfunction at least a month after exposure to a traumatic event^1^. PTSD after delivery was described in literature since the 1970s^2^,^3^,^4^,^5^,^6^,^7^,^8^. Studies suggest that not only severe gynaecological pathology during delivery and a negative outcome can be a traumatic experience for the mother, but that normal delivery (at the expected time with a healthy child) can also cause PTSD^3^,^4^,^5^.

Prevalence of postpartum diagnosis of PTSD varies in the range of 0–6.9% (2% on average), but significant levels of symptoms of the subthreshold PTSD could be present in 1.5–33.1% of all parturients^3^,^9^,^10^,^11^,^12^,^13^.

Postpartum PTSD has a significant influence on mother and baby’s daily and long-term functioning, a high comorbidity rate with other mental disorders, but remains rarely recognised and treated in routine clinical practice. Consequences of postpartum PTSD include: refusal to breastfeed the new-born, attachment problems, partner and intimacy avoidance, low self-esteem and low self-adequacy. These women associate their delivery to pain, fear or sorrow, or have traumatic amnesia^5^. It is unclear which biological and psychological risk factors are crucial for the development of postpartum PTSD. This indicates the urge for research in this field.

Risk factors for PTSD after delivery are mostly the same as for PTSD after any other trauma, with some specificity related to the event^14^, but not much is known about specific risk factors for PTSD after regular vaginal delivery. These risk factors might be pre-traumatic, peritraumatic and posttraumatic.

Possible pre-traumatic factors include sociodemographic factors^5^,^6^,^15^, personality traits^5^,^7^, tocophobia^13^, history of previous psychological problems^4^,^10^,^13^,^14^,^15^,^16^ or the number of previous deliveries^9^,^15^,^16^. Risk factors related to delivery include: mode and duration of delivery^6^,^9^,^12^,^17^, obstetric factors and complicated deliveries^6^,^14^,^16^, pain^5^,^9^ and epidural analgesia^9^,^18^, intensive fear for own or baby’s life^4^,^5^,^15^, sleep disturbances^19^ or peripartal dissociation^13^. Certain factors can be related to pre-, peri- and postpartum period (e.g. lack of social and medical support or traumatic experiences)^3^,^4^,^5^,^13^,^14^,^15^. Physiological measures as heart rate and blood pressure levels or low cortisol levels as parameters of sympathetic activation have been also suggested as potential risk factors for consecutive PTSD^20^,^21^,^22^.

Many of the listed risk factors are not studied together in the population of primiparous women with vaginal delivery. The study of postpartum PTSD requires a multidimensional approach since this disorder is a result of synergy of multiple risk factors, including biological vulnerability, psychological experiences and social influences.

The objectives of our study were to determine the prevalence of postpartum PTSD and to evaluate possible risk factors. The present study explored different risk factors: sociodemographic, obstetrical procedures, applied medications, epidural analgesia, biological (heart rate and blood pressure), and social support (partner’s presence) for postpartum PTSD in a unique population of primiparous women with vaginal delivery. This is the first study, as far as we are aware, on postpartum PTSD in Southeast Europe.

## Methods

### Participants

The study included 150 women who were recruited consecutively according to eligibility (inclusion and exclusion criteria) and was carried out at the Institute of Mental Health in Belgrade and the University Clinic of Obstetrics and Gynaecology “Narodni Front” from 2011–2012. The study received approval of the Ethical Committees of both institutions. Methods were carried out in accordance with the approved guidelines. All the participants signed the informed consent and were eligible if they were primiparous aged 18 or over, with or without any specific traumatic event until the delivery term (37–40 weeks of gestation). All of them were citizens of Belgrade. The exclusion criteria were the following: *in vitro* fertilisation pregnancy; weight below 45 or above 100 kg; psychoactive substance abuse; a prior history of PTSD and treatment for it; history of psychiatric disorders; history of head injury (involving confusion, loss of consciousness or amnesia); medical history of Cushing’s syndrome, current infectious disease or diabetes; taking medications that can interfere with the HPA axis during pregnancy or delivery (steroids, beta blockers, indomethacin); exposure to trauma that reflects ongoing victimisation (domestic violence) to which the subject was likely to be re-exposed during the study period. Women who had caesarean section were not eligible for the study.

Eight women refused to participate in the study due to “blood sampling fear”. Of the 150 parturients, one was excluded because of damaged serum sample due to sample transportation problems. One hundred and twenty-six women completed the one month follow-up interview, and seventy seven (51.3%) parturients completed the whole study. This rate of participants’ drop out does not differ from previously published data in PTSD studies in which it was estimated to be 50%^23^. No difference was found between those who remained in the study and those who withdrew regarding age (t = −0.786; p = 0.433), length of marriage (Z = −0.052; p = 0.959), educational level (Х^2^ = 2.834, р = 0,105), or frequency of epidural analgesia (Х^2^ = 0.000, р = 1.000).

The data about drug treatment during labour and delivery were collected. In accordance with hospital protocol, oxytocin (Syntocinon) was given to women as continuous intravenous drip. Epidural analgesia was applied at the time of cervix dilatation of 3–5 cm or depending on intensity and frequency of contractions (contractions at 2–5 minutes and lasting 45–60 seconds). Evaluation of specific obstetrical traumatic experience was based on objective measurements and included forceps delivery, vacuum extraction and breech presentation of the foetus.

### Procedures

The interviews were conducted during the home visits or at the Institute of Mental Health during three months after delivery.

### Cardiovascular measures

Blood pressure was measured during the first two hours after delivery. After measuring women’s heart rate (beats per minute) by a trained nurse, blood pressure (mmHg) was measured while the parturient was still lying and had rested for at least five minutes.

### Psychological measures

During the next three months, women were screened monthly for PTSD by Clinician-Administered PTSD Scale (CAPS)^24^. Traumatic experience of delivery was assessed by the A criteria of the DSM IV classification (APA, 1994), according to work of Alcorn *et al.*^25^. In CAPS, the symptoms of clusters B, C and D are rated for both frequency and intensity and these two scores are summed up to provide severity ratings. Additional questions are used to assess criteria A, E and F. A symptom was considered present if frequency was recorded as ≥1 and intensity as ≥2. PTSD diagnosis was made if a patient had at least 1 positive item from B cluster, 3 from C and 2 from D cluster and the total score above 45. Partial PTSD was diagnosed if the CAPS total score was ≥19^25^. In the reliability test, Cronbach alpha coefficient was 0.793 indicating that the CAPS has good internal consistency.

We estimated the following risk factors: sociodemographic, history of previous psychological problems, obstetrical factors and complicated deliveries, epidural analgesia, applied medications, cardiovascular measures (heart rate and blood pressure) and social support (partner’s presence). The data about the risk factors were collected by the clinical interview, from the medical records, by blood pressure measuring and with applied PTSD scale.

### Statistical analysis

Data are presented as mean ± SD or N (%). The Chi-squared test was used to compare categorical variables, while the t-test and Mann-Whitney U-test were used for comparisons of continuous data. The linear mixed model was used for longitudinal data analysis. Data were analyzed using SPSS 20.0 (IBM, corp.) statistical package. All p-values less than 0.05 were considered significant.

## Results

### Sample characteristics

Sociodemographic and clinical characteristics of the sample are presented in Tables 1 and 2. One hundred and two (81.6%) women were married with the mean length of marriage of 20.1 months (SD = 32.3) and there were no divorced women or widows.

Comparing the data of all delivered women in Belgrade during 2012, obtained from the Institute of Public Health of Belgrade with our sample, there was no difference regarding age (p = 0.178) (27). The main difference was that obstetrical interventions were more frequent in our sample (p < 0.001).

Nine (7.1%) parturients received the pain killer Dolantin (meperidine hydrochloride), a synthetic opioid analgesic, in a dosage of 50 mg intramuscular (i.m.), once during the delivery. Also, nine (7.1%) women received 10 mg i.m. of diazepam once during labour. Epidural analgesia as a cocktail of fentanyl and bupivacaine hydrochloride was applied to 90 (71.4%). The obstetrical traumatic experience occurred in 12 (9.5%) women; forceps delivery was applied in 1 (0.8%), vacuum extraction in 10 (7.9%) women, and breech presentation of the foetus in 1 (0.8%) delivery.

### Prevalence of PTSD

The full PTSD was diagnosed in 3 (2.4%) parturients after the first month of delivery, and after the next two months there were no parturients with this diagnose. The partial PTSD was found in 15 (11.9%) women one month after delivery, 5 (5.9%) after two months and one (1.3%) after three months. We analysed parturients with full and partial PTSD together as the “PTSD group”. After the first assessment there were no new cases of parturients with PTSD symptoms.

The severity of intrusive, avoidance and hyperarousal PTSD symptoms in the whole sample during the three months since delivery are presented in Table 3. After the first month, the majority of parturients (66.7%) had mild PTSD according to CAPS, and none of them had an extremely high score of PTSD symptoms. We have found that 64.3% of the parturients with PTSD symptoms after the first month did not have the symptoms after the second month, and 92.9% of them recovered after the third month.

**Figure 1:**
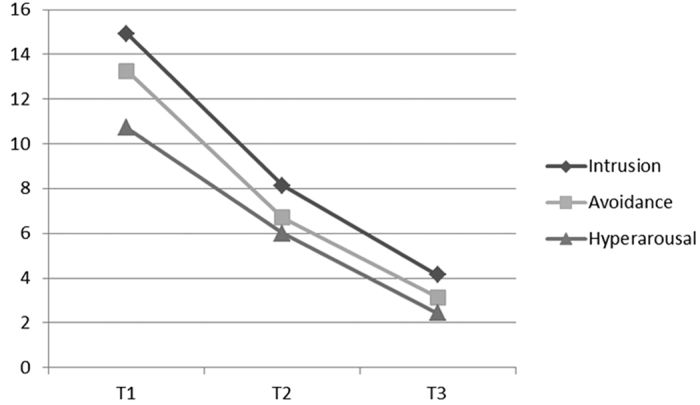
Intensity of PTSD symptoms decreased during the three months of follow up after delivery.

An analysis by the linear mixed model of parturients with PTSD symptoms has shown that the total PTSD symptoms significantly decreased (F = 89.141, р < 0.001) during the three months of follow up, the same as the intrusive (F = 56.934, p < 0.001), avoidance (F = 64.795, p < 0.001) and hyperarousal (F = 138.845, p < 0.001) symptoms (Fig. 1).

### Risk factors for PTSD

There were no significant differences for the sociodemographic data or clinical characteristics of the sample between the PTSD group and the parturients without PTSD symptoms. *P* varied from 0.152–1.000. Only the variable “being married” showed a trend to be significant (Z = −1.767, p = 0.077) (Tables 4 and 5).

The most important finding is that the PTSD group of parturients had significantly more obstetrical interventions during delivery (Х^2^ = 50.346, р < 0.001). The univariate analysis has shown that the only statistically significant predictor of PTSD were obstetrical interventions (X^2^ = 50.436, p < 0.01). Multivariate binary logistic regression confirmed statistically significant relation of obstetrical interventions and PTSD, while no other predictors were significant (OR 53.5; Adj OR = 144.8).

## Discussion

Women can experience delivery as traumatic and develop PTSD which is neglected both by gynaecologists and psychiatrists. The objective of our study was to evaluate the prevalence of postpartum PTSD and potential risk factors for the disorder in a unique sample of primiparous women with vaginal delivery. Our main findings show that 2.4% of parturients developed full PTSD after the first month since delivery and that specific obstetrical traumatic experiences during delivery are a risk factor for postpartum PTSD. No other sociodemographic variable, applied medication or epidural analgesia were significantly connected to development of PTSD symptoms.

The majority of delivered women in our study have not experienced their delivery as traumatic–only 15 of 126 (11.9%) did so after the first month and all of them had some symptoms of PTSD. Our findings of 2.4% parturients with full PTSD, after the first month of delivery, are in line with the previously published data on prevalence of postpartum PTSD^3^,^9^,^10^,^11^,^12^,^13^. Both full and partial PTSD in our sample had a decrease in intensity in the first three months which confirms the assumption that most people spontaneously recover after exposure to trauma^27^,^28^ although some authors suggest that symptom severity decreases in patients with partial PTSD while it persists in patients with full PTSD^29^. Cultural differences could influence the prevalence of PTSD, along with stigma of mental health issues. As an example of this we would like to stress out that in our sample only 4% of partners were present during delivery which significantly differs from the percent in, for instance, England^30^. It could be discussed if this finding suggests that women in our sample lack social (partner’s) support during labour and delivery (social support has been known to be an important protective factor for preventing PTSD)^31^.

The findings of obstetrical interventions as risk factors for PTSD symptoms are previously recognized in several studies^6^,^8^,^14^,^29^. Olde^12^ and Söderquist *et al.*^9^ found that emergency caesarean section (EmCS), forceps delivery, vacuum extraction, epidural analgesia and episiotomy are predisposing factors for PTSD symptoms.

Although the experience of intensive pain is correlated with negative assessment of delivery, up-to-date pain-relief techniques did not show expected results^18^. Studies suggest that women who request epidural analgesia are more anxious and have more probability to experience delivery as traumatic^9^. In a randomized controlled trial Howell *et al.* found no different effects of epidural analgesia on women’s birth experience^18^. In our sample, both groups of parturients (with and without PTSD symptoms) had epidural analgesia equally frequently and so it was not recognised as a risk factor for the development of the PTSD symptoms. Indirectly, we could assume that the PTSD group was not more anxious during labour or delivery (used neither anxiolytics nor epidural analgesia more often).

A limitation of our study is small sample size. In further research, a large sample drop out must be taken into consideration. As a result of small numbers, some differences between groups could not be analysed and possible correlations were not registered. It is possible that the discrepancy between the expected and perceived level of pain is an additional risk factor for traumatic experience^5^,^7^ and women’s pain should be measured in correlation with the length of delivery and potential blood loss^6^,^17^. During the postpartum period, some healthy women have numerous symptoms (fatigue, sleep disturbance, concentration problems etc.) which could easily be attributable to PTSD, so specific attention should be paid when evaluating these symptoms. However, our study has several strengths. We have used the CAPS scale which is the gold standard interview for PTSD diagnosis and thus avoided possible misdiagnose made by self-report scales^14^. Our study is a prospective and we included only primiparous women who were prepared for normal vaginal delivery (NVD), although some were delivered with instrumental vaginal delivery (IVD). Sociodemographic data, data on pre-traumatic experiences and personal medical history were collected at the moment of entering the delivery room, before knowing the final mode of delivery. Knowing that the most stressful mode of delivery for women is shown to be IVD, then EmCS, NVD and elective cesarean section^9^,^13^, we tried to refine our sample and show that NVD *per se* could be the source of PTSD. According to the fact that parturients with a prior history of PTSD and treatment for it were not eligible for the study, trauma in our study was specifically related to labour and delivery, which probably reduced the occurrence of postpartum PTSD.

Although not the first study to explore the relationship between delivery and PTSD, our study is the first in Southeast Europe to include only NVD primiparous women and our findings show that NVD is not a risk factor for PTSD. Our study is the first in Southeast Europe to include only NVD primiparous women and our findings show that NVD is not a risk factor for PTSD. The obstetrical complications as an unplanned change significantly increase the likelihood of PTSD. Personality traits, previous stressful experiences, and exposure to traumatic events may have an independent and direct influence on developing posttraumatic stress^32^, and according to this we suggest that recently delivered women who had obstetrical complications should be screened and treated for PTSD as soon as possible. It has been shown that when treatment is applied early (e.g. cognitive behavioral therapy), during the first month, it can reduce symptoms of PTSD quicker^33^, which is especially important due to the fact that mother-child bonding is very strong in that period. Implications of our findings could be in the revealing of possible risk factors and vulnerability of parturients with PTSD, which could help professionals to better tailor the prevention measures for PTSD. A close collaboration between gynecologists, psychiatrists and primary care physicians is needed.

## Additional Information

**How to cite this article**: Milosavljevic, M. *et al.* Posttraumatic Stress Disorder after Vaginal Delivery at Primiparous Women. *Sci. Rep.* **6**, 27554; doi: 10.1038/srep27554 (2016).

## Figures and Tables

**Table 1:** Age and clinical characteristics of the sample.

	mean ± SD (Min-Max)
Age	29.7 ± 4.0 (20.0–42.0)
Weight (kg)	75.2 ± 9.0 (53.0–99.0)
Heart rate (n/min)	78.7 ± 10.9 (56.0–110.0)
Systolic blood pressure (mmHg)	114.0 ± 10.8 (90.0–150.0)
Diastolic blood pressure (mmHg)	71.5 ± 8.4 (60.0–100.0)

Legend: kg- kilogram; n/min-number/minute; mmHg- millimeter of mercury.

**Table 2:** Sociodemographic characteristics of the sample and applied medications.

	Category	n	%
Education	Elementary school	1	0.8%
	High school	56	44.4%
	College	14	11.1%
	University degree	55	43.7%
Marital status	Single	6	4.8%
	Married	102	81.6%
	Common-law marriage	17	13.6%
Living with partners	No	6	4.8%
	Yes	119	95.2%
Employed	No	29	23.0%
	Yes	97	77.0%
Family history	No	115	91.3%
	Yes	11	8.7%
Applied pain killers	No	117	92.9%
	Yes	9	7.1%
Applied anxiolytics	No	117	92.9%
	Yes	9	7.1%
Epidural analgesia	No	36	28.6%
	Yes	90	71.4%
Partner was present during labour	No	121	96.0%
	Yes	5	4.0%

**Table 3:** Descriptive statistics of the clusters of PTSD symptoms (CAPS score) after 1, 2 and 3 months of delivery.

Symptoms	Month		
	1^st^	2^nd^	3^rd^
Intrusive	14.9 ± 5.6 (8.0–26.0)	8.1 ± 3.5 (4.0–17.0)	4.1 ± 3.5 (0.0–12.0)
Avoidance	13.3 ± 5.4 (4.0–22.0)	6.7 ± 4.3 (0.0–15.0)	3.1 ± 2.3(0.0–8.0)
Hyperarousal	10.7 ± 3.1 (4.0–16.0)	6.0 ± 2.6 (2.0–10.0)	2.4 ± 1.9 (0.0–6.0)
Total	38.9 ± 12.4 (20.0–60.0)	20.9 ± 8.9 (10.0–39.0)	9.9 ± 6.7 (3.0–26.0)

Data are presented as mean ± SD (Min-Max).

**Table 4:** Sample details of the parturients with and without PTSD symptoms.

		PTSD symptoms		t/Z/X^2^	p
		Yes	No		
Age		28.93 ± 3.63	29.86 ± 4.10	−0.827	0.410
Type of partnership	single	2 (13.3%)	4 (3.6%)	3.194	0.183
	married	12 (80.0%)	90 (81.8%)		
	common-law marriage	1 (6.7%)	16 (14.5%)		
Living with partner	Yes	13 (86.7%)	106 (96.4%)	2.716	0.152
	No	2 (13.3%)	5 (3.6%)		
Educational level	Primary school	0 (0.0%)	1 (0.9%)	0.457	0.572
	High school	5 (33.3%)	51 (45.9%)		
	College	3 (20.0%)	11 (9.9%)		
	University degree	46 (74.2%)	52 (81.3%)		
Employment	Yes	12 (80.0%)	85 (76.6%)	0.087	1.000
	No	3 (20.0%)	26 (23.4%)		
Family history	Yes	1 (6.7%)	10 (9.0%)	0.091	1.000
	No	14 (93.3%)	101 (91.0%)		
Applied pain killers	Yes	1 (6.7%)	8 (7.2%)	0.006	1.000
	No	14 (93.3%)	103 (92.8%)		
Applied anxiolytics	Yes	1 (6.7%)	8 (7.2%)	0.006	1.000
	No	14 (93.3%)	103 (92.8%)		
Epidural analgesia	Yes	10 (66.7%)	80 (72.1%)	0.189	0.762
	No	5 (28.6%)	31 (27.9%)		
Partner’s presence	Yes	–	5 (4.5%)	0.704	0.632
	No	15 (100%)	106 (95.5%)		
Obstetrical interventions	Yes	9 (75.0%)	3 (25.0%)	50.346	<0.001
	No	6 (5.3%)	108 (94.7%)		

Legend: The data are presented as n (%) or mean ± SD; *t*-t-test; *X*^*2*^-Chi-squared test; *Z-* Mann-Whitney U-test.

**Table 5:** Clinical characteristics of the parturients with and without PTSD symptoms.

	PTSD symptoms		t	p
	Yes	No		
Weight (kg)	73.87 ± 10.90	75.41 ± 8.81	−0.617	0.539
Systolic blood pressure (mmHg)	111.33 ± 7.18	114.37 ± 11.22	−1.419	0.169
Diastolic blood pressure (mmHg)	70.67 ± 7.76	71.62 ± 8.56	−0.410	0.683
Heart rate (n/min)	79.93 ± 8.55	78.56 ± 11.23	0.456	0.649

Legend: The data are presented as mean ± SD; t-t-test; p-sign; kg- kilogram; n/min-number/minute; mmHg- millimeter of mercury.
